# Motivating Factors for Continuous Participation in a Municipality‐Initiated Incentive‐Based Health Promotion Program: A Qualitative Study

**DOI:** 10.1002/jgf2.70117

**Published:** 2026-05-01

**Authors:** Hana Wakasa, Isaku Kurotori, Michiko Aoyanagi, Takashi Kimura, Akiko Tamakoshi

**Affiliations:** ^1^ Department of Public Health, Faculty of Medicine Hokkaido University Sapporo Japan; ^2^ School of Nursing Sapporo City University Sapporo Japan; ^3^ Health and Environmental Risk Division National Institute for Environmental Studies Tsukuba Japan

**Keywords:** health promotion, incentive, Japanese, qualitative study

## Abstract

**Background:**

Noncommunicable diseases (NCDs) are the leading global cause of death, with physical inactivity being a major modifiable risk factor. In Japan, high NCD mortality and low physical activity rates prompted municipalities to launch incentive programs promoting healthier behaviors. However, long‐term experiences and motivations beyond immediate rewards remain unclear.

**Methods:**

A qualitative descriptive study was conducted in Nakasatsunai Village, Hokkaido, Japan. In 2022, 17 individual and one focus group interviews were held with 21 participants continuously engaged in the Health Point Project. Participants earned points through step tracking, health checkups, and events, which could be exchanged for vouchers. Data were analyzed using inductive content analysis with co‐author review and member checking.

**Results:**

Participants' mean age was 60.3 years. Eight categories described continued participation: looking forward to the benefits without active effort; being encouraged to engage in physical activity within well‐designed environments; hoping to maintain good health; recognizing the benefits and impacts of exercise; engaging in activities in their own way or style; adopting exercise and health care as habits; being inspired by peers; and feeling a sense of community. Incentives motivated initial participation, while habit formation, health improvements, social interaction, and community involvement sustained long‐term engagement.

**Conclusions:**

Participants' sustained involvement was driven not only by financial incentives but also by experiences of personal connection and community belonging. Thus, municipal incentive programs may enhance both individual health behaviors and social well‐being, underscoring the importance of integrating opportunities for social interaction alongside financial incentives in sustainable health promotion initiatives.

## 1 Introduction

According to the World Health Organization, noncommunicable diseases (NCDs) cause 41 million deaths each year, accounting for approximately 74% of all global deaths [1]. Physical inactivity is one of the major risk factors for developing NCDs and imposes a substantial economic burden, with treatment costs exceeding US$300 billion annually [2]. In Japan, NCDs and physical inactivity are also major public health concerns; NCDs account for 85% of total deaths, and about 35% of adults aged 18 years and older are physically inactive [3]. To address these challenges, the Japanese government launched the Heath Japan 21 (Third Term) policy in 2023 [4]. The policy aims to increase the average number of daily steps, raise the proportion of individuals with regular exercise habits, and reduce the number of children who do not participate in regular physical activity or sports.

Incentive‐based approaches that encourage individual behavioral change to promote health have recently gained global attention [5]. A systematic review revealed that financial incentives can promote healthier dietary choices, reduce alcohol consumption, and encourage weight loss and physical activity associated with NCD prevention [6]. Several systematic reviews and meta‐analyses have provided evidence supporting the effectiveness of incentives in changing health behaviors, including physical activity and exercise habits [7, 8, 9]. Although monetary rewards have been shown to be the most effective for promoting initial program enrollment [10], there is no standardized method for implementing incentives. Feasible and sustainable programs should therefore be tailored to the specific context of each region. Moreover, the factors influencing sustained participation during long‐term or post‐intervention periods in incentive programs remain unclear.

In Japan, the Nippon Kenko Kaigi was established in 2015 as a collaborative initiative between local governments and private organizations to expand advanced preventive and health promotion efforts nationwide among insurers and other stakeholders. The Healthy Cities and Workplaces Declaration 2020, a policy of the Nippon Kenko Kaigi launched in 2015, aimed to promote incentive‐based health initiatives targeting the general population across more than 800 municipalities [11]. In 2019, the Ministry of Health, Labour and Welfare introduced the Health Life Extension Plan to promote health‐related incentives and extend healthy life expectancy [12]. The government also recommended that insurers provide incentives as part of their mandatory efforts under the National Health Insurance Act and related regulations, and accordingly formulated relevant guidelines [13, 14]. As a result, by 2020, 1024 municipalities had implemented health promotion incentive programs [15]. The next step is to assess whether these initiatives effectively promote behavioral change and habit formation, rather than merely encouraging participation for its own sake.

Previous Japanese studies have demonstrated the positive effects of municipality‐initiated incentive programs. One study reported that incentive‐based health promotion programs implemented in six municipalities increased physical activity among previously inactive residents 18 months after participation [16]. Another study found that participants who remained in the maintenance stage for 6 months or longer after changing their health behaviors were those who had achieved pre‐determined incentive targets [17]. A further study showed that participants in an incentive program preferred cash or cash‐equivalent rewards over community‐based rewards, as these were more effective in preventing program dropout during a 1‐year observation period [18]. However, no studies have examined participants' experiences related to long‐term motivational maintenance beyond the influence of incentives. Understanding motivations that extend beyond financial rewards is essential for sustaining engagement even after incentive programs conclude. Therefore, this qualitative study explored individuals' experiences of continuous participation in a local incentive‐based program over several years.

## 2 Method

### 2.1 Design

This study employed a qualitative descriptive design [19] to explore the experiences of participants who had continuously taken part in an incentive program implemented in a Japanese municipality. The study was reported in accordance with the Consolidated Criteria for Reporting Qualitative Research [20]. Ethical approval was obtained from the Ethical Review Board for Life Science and Medical Research, Hokkaido University Hospital (Approval no. 023‐057).

### 2.2 Setting

Seventeen individual semi‐structured interviews (30–60 min each) and one 90‐min focus group interview were conducted on June 27–28 and July 3, 2022, with individuals who had continuously participated in the Health Point Project in Nakasatsunai Village, located in eastern Hokkaido, Japan. The village had a population of 3838 at the end of fiscal year 2021. The pilot program began in fiscal year 2017 and was fully implemented in fiscal year 2018. Participants earned points through various activities, including step‐count tracking, blood pressure measurement, health checkups, and participation in village‐designated events. In the following fiscal year, the program was expanded to include two participation courses: the Basic Course and the Challenge Course. The Basic Course followed a structure similar to the previous year's program, allowing participants to exchange points for vouchers worth up to 1000 yen (≈ US $6.79 as of October 3, 2025), redeemable at local establishments. The Challenge Course required a 1000‐point participation fee but allowed participants to exchange their accumulated points for vouchers worth up to 5000 yen, also usable within the village. Additionally, Challenge Course participants could donate up to another 5000 yen to local schools, with total awarded points amounting to a maximum equivalent of 10,000 yen. During fiscal year 2022, 552 eligible individuals participated in the Challenge Course.

### 2.3 Sampling and Recruitment

To enhance the rigor and trustworthiness of the study [21, 22], participants who had actively and continuously taken part in the project during fiscal year 2022 were selected and invited to participate in interviews by staff members from the village office. Each participant received a 500‐yen gift certificate as compensation for participation. Participants were informed about the anonymity and confidentiality of the discussions, their right to withdraw from the study at any time, and the intended publication of the findings. Written informed consent was obtained from all participants.

### 2.4 Measures

Semi‐structured interviews, consisting of one focus group interview and 17 individual interviews, were conducted in private rooms at the village health and welfare center, which was easily accessible to residents. Recognizing the sensitivities of the small, close‐knit community, we allowed participants to choose between individual interviews or a focus group based on their personal preference and comfort level to ensure psychological safety and minimize social desirability bias. The interviews were conducted by the first author (female, MPH, RN), the second author (male, PhD, MD, psychiatrist), and the fourth author (male, PhD, epidemiologist), all of whom specialize in public health. Participants first provided basic demographic information, including their name, age, sex, family structure, length of residence in the village, and occupation. All interviews were conducted in Japanese, using an interview guide (Table 1) developed with consideration of participant characteristics to achieve the study's objectives. The interview guide was developed under the guidance of an expert experienced in qualitative research and was further reviewed by municipal staff members familiar with the local context to ensure face validity and cultural appropriateness. Prior to the formal study, a pilot test was conducted with individuals matching the target demographic to refine the clarity and flow of the questions. After completing the interviews, recurring patterns and preliminary themes were identified, and data saturation was considered achieved.

**TABLE 1:** Interview guide.

No.	Questions (translated into English from the original Japanese interview guide)
1	How do you feel about your participation in this program so far?
1–1	What prompted you to join the program? Was there a specific reason you would like to share?
1–2	Do you have any friends or acquaintances who are also participating in this program?
2	Have you noticed any benefits from participating in this program?
3	In what ways has your life changed as a result of participating in this program?
4	What motivates you to continue participating in this program?
4–1	If you have ever considered quitting, what made you decide to stay?
5	Are you aware of anyone who is still actively participating in this program?
5–1	What do they think about the program?
6	Do you know anyone who has quit participating in this program?
6–1	What do they think about the program?
7	If you have ever talked with others about this program, could you please describe those conversations?

### 2.5 Data Analysis

To explore participants' ongoing experiences, the recorded data were analyzed using inductive content analysis. Data from both individual interviews and the focus group were combined and analyzed as a single dataset to identify common recurring themes across different conversational settings. This integrated approach allowed for a comprehensive understanding of the community's shared perspectives while accounting for various levels of participant interaction. The first and second authors conducted the analysis following these steps: (a) the verbatim transcripts were repeatedly read to grasp the overall context; (b) each sentence relevant to the study aim was classified into open codes; (c) similar codes were grouped into subcategories; and (d) broader categories were developed to represent more comprehensive concepts. The four co‐authors assessed and reviewed the codes, subcategories, and categories. Data analysis was conducted in Japanese, and the results were later translated into English. The authors discussed and refined the findings collaboratively until consensus was achieved. In addition, a member‐checking process was conducted with two participants and one village officer who was familiar with the participants' situation to confirm the credibility of the results.

## 3 Results

### 3.1 Characteristics of Participants

A total of 22 participants took part in semi‐structured interviews: five participated in a focus group, and 17 took part in individual interviews. One woman participant from the individual interview group was excluded from analysis because she appeared to have confused this project with another one. Thus, data from 21 participants (nine men and 12 women) were included in the analysis. Participant characteristics are presented in Table 2. The mean age of the participants was 60.3 years (standard deviation [SD] = 15.8). Nineteen participants (90.5%) were enrolled in the Challenge Course. Even the participant with the shortest engagement had participated for 10 months. Considering that the maintenance stage in the Transtheoretical Model is defined as a period of at least 6 months following behavioral change, all participants were considered to have engaged in long‐term participation.

**TABLE 2:** Characteristics of Participants.

No.	Sex	Age group (years)	Type of interview	Course	Continuous participation duration (months)
1	Woman	75—	One‐to‐One	Challenge	48
3	Woman	75—	Focus Group	Challenge	47
4	Woman	75—	Focus Group	Challenge	49
5	Woman	65–74	Focus Group	Challenge	49
6	Woman	65–74	Focus Group	Challenge	59
7	Woman	65–74	Focus Group	Challenge	48
8	Woman	45–64	One‐to‐One	Challenge	47
10	Woman	65–74	One‐to‐One	Challenge	37
11	Woman	65–74	One‐to‐One	Challenge	46
12	Man	18–44	One‐to‐One	Basic	36
13	Man	45–64	One‐to‐One	Basic	35
14	Man	18–44	One‐to‐One	Challenge	36
15	Man	45–64	One‐to‐One	Challenge	12
16	Man	18–44	One‐to‐One	Challenge	47
17	Man	18–44	One‐to‐One	Challenge	50
19	Man	45–64	One‐to‐One	Challenge	47
21	Woman	75—	One‐to‐One	Challenge	37
22	Woman	65–74	One‐to‐One	Challenge	49
24	Man	18–44	One‐to‐One	Challenge	50
25	Woman	45–64	One‐to‐One	Challenge	13
27	Man	65–74	One‐to‐One	Challenge	10

### 3.2 Experiences of Continued Participants

Eight categories were identified to represent participants' experiences of continued engagement: looking forward to the benefits of participation; being encouraged to engage in physical activity within well‐designed environments; hoping to maintain good health; recognizing the benefits and impacts of exercise; engaging in activities in their own way or style; adopting exercise and health care as a habit; being inspired by other participants; and feeling a sense of community. Twenty‐three subcategories were also derived (Table 3).

**TABLE 3:** Experiences that motivated long‐term participation.

Category	Subcategory
Looking forward to the benefits without active effort	Being motivated by points and vouchers
	Anticipating program events with interest
	Gaining health‐related knowledge
Being encouraged to engage in physical activity within well‐designed environments	Feeling a valid reason to promote health and exercise
	Experiencing little inconvenience or burden in participation
	Having easy access to walking and exercise opportunities
Hoping to maintain good health	Seeking to overcome health issues
	Taking proactive measures to protect one's health
	Aiming to maintain good health and stay active in older age
Recognizing the benefits and impacts of exercise	Paying attention to and assessing measured health values
	Finding enjoyment in physical activity
	Noticing positive physical changes
Engaging in activities in their own way or style	Aligning participation with existing habits or needs
	Setting individualized step goals
	Implementing personal strategies to incorporate exercise into daily routines
Adopting exercise and health care as habits	Integrating walking and exercise into daily life
	Striving to walk or move more every day
	Becoming aware of healthy eating habits
Being inspired by peers	Joining the program after encouragement from friends or acquaintances
	Engaging in friendly comparison or competition with others
	Feeling inspired by fellow participants
Feeling a sense of community	Developing interest and pride in the local community
	Increasing communication and interaction among residents

### 3.3 Looking Forward to the Benefits Without Active Effort

Most participants mentioned that earning points and receiving vouchers motivated them to continue participating in the program.

Accumulating points and exchanging them for vouchers is quite motivating. I think the best part is that accumulating points leads to a tangible reward in the form of vouchers. I am not just happy to receive something for doing activities, but rather, I appreciate that the voucher amount increases based on my effort. I prefer receiving vouchers to other items. (Participant 16)

Normally, I don't like getting on the scale, but I started doing it because I can earn points. (Participant 17)

Participants also expressed anticipation for program events and appreciated opportunities to gain health‐related knowledge. Access to these activities and information enhanced their motivation to stay engaged.

The special walking events increase my motivation to participate. For example, in the ‘Hawaii Edition’ event, famous sightseeing spots were introduced along the walking route, which made it enjoyable. (Participant 8)

Attending a nutritionist's lecture related to the program allowed me to learn a lot, which was beneficial. (Participant 10)

### 3.4 Being Encouraged to Engage in Physical Activity Within Well‐Designed Environments

Participants mentioned that the program itself served as a motivation to take care of their health.

I think many residents have found a sense of purpose through the program. Walking used to be seen as something mainly for older people. But now, since everyone is participating, it provides a sense of legitimacy—or a valid reason—to exercise. Residents now feel that engaging in physical activity is natural. Although many previously preferred to walk where they wouldn't be seen, they now walk more confidently than before. (Participant 17)

In addition, participants reported that they experienced little inconvenience in participating and found the walking environment comfortable.

I can gain points just by walking; it's simple and allows me to continue easily. (Participant 14)

In the village, many residents know each other, and some used to feel self‐conscious about being seen exercising and wanted to keep it private. However, I believe they no longer hesitate to exercise in public. (Participant 17)

### 3.5 Hoping to Maintain Good Health

Participants joined the program when they felt unwell, experienced health issues, or were identified as having problems during health examinations. They aimed to maintain good health and prevent illness through regular activities and viewed the program as a valuable means of achieving these goals.

My muscle strength had decreased, and although I knew how to improve it, I didn't have an exercise routine and couldn't do it. Walking was the easiest activity, and everyone was doing it. (Participant 11)

I am participating in this program to stay healthy. It helps me stay committed. (Participant 22)

In addition, most older participants expressed a desire to remain healthy throughout their lives, being mindful of the potential burden on themselves and their families in the future.

I want to avoid becoming bedridden and not be a burden on my family. Even if I eventually need care, I try to stay physically active to keep that period as short as possible. (Participant 3)

### 3.6 Recognizing the Benefits and Impacts of Exercise

Participants expressed interest in observing physical changes through the measurement of their physical parameters and activity levels.

I find it helpful to track my weight and grip strength each week and see how they change. (Participant 27)

They also described feelings of joy and satisfaction arising from noticeable improvements in their bodies and family relationships.

When I started the program, I weighed 90 kg, but I have lost about 5 kg since then. (Participant 21)

I started walking more, such as taking walks with my child. I have realized that playing outside with my child is also beneficial for me. (Participant 17)

### 3.7 Engaging in Activities in Their Own Way or Style

Participants mentioned that the program aligned naturally with their existing habits.

I originally had a habit of walking with my dog, and that habit naturally connected to this program. (Participant 3)

Additionally, participants found ways to set personal goals and incorporate exercise into their daily lives without overexerting themselves, often through creative approaches.

I try to walk as much as possible—for example, by avoiding the use of a car when shopping and staying active even inside the house. (Participant 10)

Since I can't keep up with younger people, I walk at my own pace. Gateball and park activities require gathering teammates and adjusting schedules, but I prefer to walk whenever I have free time. (Participant 1)

I used to push myself really hard to get 10,000 steps every day, but lately I have been taking a more relaxed approach—just aiming to reach 10,000 by the end of the day. (Participant 10)

### 3.8 Adopting Exercise and Health Care as Habits

Participants mentioned that they had developed habits of engaging in physical activities and had integrated these routines into their daily lives.

After dinner, I walk every day, but only in nearby, well‐lit areas. (Participant 1)

I feel happy when my family tells me I look energetic because of participating in the program. My husband and I have discussed it and agreed that we will continue exercising even if this program is discontinued. (Participant 10)

I used to spend a lot of time sitting on the sofa on my days off, but now I make an effort to go for walks. (Participant 15)

Participants also shared that they try to take additional steps whenever possible.

I have started to catch myself thinking I should take the stairs instead of the elevator. (Participant 12)

When I go shopping, I try to park a little farther from the entrance so I can get in some extra steps. (Participant 14)

Moreover, participants noted that the program had increased their awareness of maintaining a healthy diet.

Seeing my step count and calories burned made me realize that no matter how much I walk, if I keep eating mostly oily foods, I will just accumulate calories. This made me more mindful of my diet. (Participant 21)

### 3.9 Being Inspired by Peers

Participants mentioned that they decided to join the program after seeing or hearing that their friends had already joined.

I knew about the program but didn't feel the need to join. Then a friend invited me to take part, so I decided to give it a try. (Participant 10)

Participants also shared that encouragement and effort from others served as important sources of motivation. After joining, many found that comparing themselves to other participants helped sustain their engagement.

When I see other participants and think, ‘Wait, they're that age already?’ it makes me feel like I should try a bit harder. (Participant 27)

We encourage each other in our group of friends, saying, ‘Let's work hard to accumulate points.’ (Participant 14)

There are so many different kinds of people in the program, so even when I am not trying that hard, I still feel encouraged. (Participant 11)

### 3.10 Feeling a Sense of Community

Participants mentioned that the program enhanced their sense of connection within the community and increased communication among residents, especially through donation‐related activities. They also noted that these changes helped strengthen both family and community bonds.

Excess points can be donated to village schools, and I stay motivated by the thought that the schools can benefit from it. (Participant 22)

When greeting other residents in the village, after talking about the weather, we naturally bring up step counts as a common topic of conversation. (Participant 17)

I started talking about step counts when meeting other residents in the village. Even when meeting strangers, we talk about walking and exchange greetings. (Participant 10)

## 4 Discussion

Using qualitative descriptive analysis, this study explored participants' experiences related to the long‐term maintenance of participation in a municipal incentive‐based health promotion program. Eight categories were identified as representing residents' experiences of active and continuous engagement. The findings revealed that while rewards such as points and vouchers initially motivated participation, various intrinsic and social factors sustained continued engagement beyond incentives. Physical activity was promoted within the structured framework of the program and supported by physical assessment tools. In addition to individual benefits such as maintaining good health, noticing physical improvements, and establishing healthy habits, the influence of other participants and enhanced communication with residents and family members emerged as distinctive features of the program. Overall, the findings suggest that the program not only improves residents' health but also fosters a community environment in which physical activity becomes more accessible and interpersonal relationships are strengthened.

Municipality‐initiated incentive programs are designed to facilitate the formation of healthy habits, and participants in this study reported that they had developed regular physical activity routines and integrated them into their daily lives. Although no previous studies have provided strong evidence supporting long‐term effects on physical activity or sustained positive behavioral changes after the completion of incentive programs [8, 23], many of these studies were short in duration—some lasting only 2 weeks—which may have been insufficient to enable habit formation. In contrast, the mean duration of participation among the current study's participants was 3.4 years, suggesting that long‐term engagement may be a key factor in sustaining behavioral change.

The results of this study indicate that participation in an incentive program not only increased physical activity but also enhanced social capital and fostered community building. Recent social epidemiological research has demonstrated that both individual‐ and group‐level social capital influence personal health. Individual‐level social capital overlaps with social support, referring to direct connections between individuals, whereas group‐level social capital reflects the indirect influence of others' behaviors within the same community [24]. A previous Japanese study found that financial incentives for physical activity among older women did not increase individual‐level social capital [25]. However, in our study, being inspired by other participants and experiencing a sense of community contributed to sustained motivation, which may help strengthen group‐level social capital. Furthermore, participants' motivation within the program may not only promote individual health but also support community development by fostering collective engagement and a shared sense of belonging. These findings suggest that such programs could play an important role in creating more socially connected and health‐conscious communities.

This study has several limitations that should be acknowledged. First, interviews were conducted solely in one village in Hokkaido, Japan, where a municipality‐initiated incentive‐based health promotion program may not represent the experiences of residents in other regions or countries. However, understanding practical and sustainable incentive programs tailored to the specific circumstances of each region remains essential. Second, participation in the qualitative interviews was voluntary, introducing the possibility of selection bias. Third, the study did not evaluate the long‐term effects on physical activity, sustained positive behavioral changes, or increases in group‐level social capital after the completion of the incentive program. Although the feasibility of program continuation is a policy matter, our findings suggest that sustained implementation of incentive programs can promote physical activity and yield broader social benefits, including strengthened community connections.

In conclusion, this study identified various motivational experiences among individuals who continued to participate in a municipality‐initiated incentive‐based health promotion program over several years. The results indicate that such programs can influence not only the formation of individual exercise habits but also the enhancement of community cohesion. These findings provide valuable insights for the design and promotion of future incentive‐based health initiatives aimed at fostering both personal well‐being and social connectedness.

## Author Contributions

**Hana Wakasa:** conceptualization, data curation, writing – original draft, formal analysis, investigation, writing – review and editing, project administration. **Isaku Kurotori:** conceptualization, writing – original draft, formal analysis, investigation, writing – review and editing. **Michiko Aoyanagi:** writing – review and editing, validation, supervision. **Takashi Kimura:** writing – review and editing, investigation. **Akiko Tamakoshi:** conceptualization, writing – review and editing, supervision, funding acquisition, project administration.

## Funding

This study was supported by joint research funds from Nakasatsunai Village.

## Ethics Statement

This study was approved by the Ethical Review Board for Life Science and Medical Research, Hokkaido University Hospital (Approval no. 023‐0057).

## Consent

Written informed consent was obtained from all participants.

## Conflicts of Interest

The authors declare no conflicts of interest.

## Data Availability

Research data are not shared.
